# 3D Convolutional Deep Learning for Nonlinear Estimation of Body Composition from Whole-Body Morphology

**DOI:** 10.21203/rs.3.rs-3935042/v1

**Published:** 2024-02-13

**Authors:** Isaac Tian, Jason Liu, Michael Wong, Nisa Kelly, Yong Liu, Andrea Garber, Steven Heymsfield, Brian Curless, John Shepherd

**Affiliations:** University of Washington; University of Washington; University of Hawaii Cancer Center; University of Hawaii - Manoa; University of Hawaii - Manoa; University of California - San Francisco; Pennington Biomedical Research Center; University of Washington; University of Hawaii - Manoa

**Keywords:** 3D Scanning, Body Composition, Machine Learning, Autoencoder, Gaussian Process

## Abstract

Total and regional body composition are strongly correlated with metabolic syndrome and have been estimated non-invasively from 3D optical scans using linear parameterizations of body shape and linear regression models. Prior works produced accurate and precise predictions on many, but not all, body composition targets relative to the reference dual X-Ray absorptiometry (DXA) measurement. Here, we report the effects of replacing linear models with nonlinear parameterization and regression models on the precision and accuracy of body composition estimation in a novel application of deep 3D convolutional graph networks to human body composition modeling.

We assembled an ensemble dataset of 4286 topologically standardized 3D optical scans from four different human body shape databases, DFAUST, CAESAR, Shape Up! Adults, and Shape Up! Kids and trained a parameterized shape model using a graph convolutional 3D autoencoder (3DAE) in lieu of linear PCA. We trained a nonlinear Gaussian process regression (GPR) on the 3DAE parameter space to predict body composition via correlations to paired DXA reference measurements from the Shape Up! scan subset. We tested our model on a set of 424 randomly withheld test meshes and compared the effects of nonlinear computation against prior linear models.

Nonlinear GPR produced up to 20% reduction in prediction error and up to 30% increase in precision over linear regression for both sexes in 10 tested body composition variables. Deep shape features produced 6–8% reduction in prediction error over linear PCA features for males only and a 4–14% reduction in precision error for both sexes. Our best performing nonlinear model predicting body composition from deep features outperformed prior work using linear methods on all tested body composition prediction metrics in both precision and accuracy. All coefficients of determination (R^2^) for all predicted variables were above 0.86.

We show that GPR is a more precise and accurate method for modeling body composition mappings from body shape features than linear regression. Deep 3D features learned by a graph convolutional autoencoder only improved male body composition accuracy but improved precision in both sexes. Our work achieved lower estimation RMSEs than all previous work on 10 metrics of body composition.

## INTRODUCTION

Total and regional body composition are correlated with many of the leading causes of death in the US and around the world. [1–4] High visceral fat deposits doubled the risk for metabolic syndrome in males with otherwise normal BMIs [4] and increased mortality rates from cancer by up to 63% [5]. Metabolic syndrome, a condition indicated by an array of biological and physical vital measurements such as blood glucose and waist circumference, is strongly associated with many chronic conditions such as cancer, heart failure, and diabetes [6–8]. Management of these conditions is also heavily impacted by body composition, specifically low lean mass, which predicts poor treatment outcomes [9, 10] and up to 17-fold increased mortality [11, 12]. However, body composition assessment historically required more advanced instruments such as dual X-ray absorptiometry (DXA) or air displacement plethysmography (ADP) [13]. Unlike ADP DXA can measure regional compartmental fat and lean deposits but exposes participants to potentially harmful ionizing radiation and is not recommended for frequently repeated measurements, particularly in at-risk groups such as young children and pregnant women. Radiation exposure must be limited to exigent circumstances even in healthy adults. An ideal alternative assessment system should achieve accuracy and precision on total and regional body composition measurement comparable to DXA without utilizing ionizing radiation. The system should be relatively inexpensive and easy to use, requiring no special training or certifications to operate and returning results in a minute or less.

Recent work showed that 3D optical (3DO) imaging can serve as an accurate and precise, low-cost, noninvasive surrogate to DXA imaging [14–16]. 3DO measures the surface geometry of the human body using light in the optical spectrum as opposed to the penetrating radiation of DXA. It does not require the injection of an isotope contrast like MRI and can scan an entire adult body in under one minute. Scanning systems cost on the order of $10,000 and are programmed by the manufacturer to operate automatically without the need for certified technicians. The external 3D shape of a human body contains strong signals about its internal structure and composition that can be learned by machine learning algorithms. Recent advances in 3D scanning technology have made 3DO scanning of human bodies more accessible and widely distributed than ever [16, 17]. However, current work on learning body composition from 3DO scans relies on linear mathematical models, such as principal component analysis (PCA) and linear regression. These simplified linear assumptions impose potentially erroneous prior assumptions on the parameterization of both the 3D shape model and the mapping between shape parameters and body composition. Nonlinear methods that relax restrictive assumptions on the estimated functions may align better with the true relationship between shape and body composition and provide better prediction accuracy of target variables. An accurate model for estimating total and regional body composition metrics from 3DO scans could standardize body composition assessment by removing the costs and risks associated with clinical evaluation and irradiation. We propose to use deep, nonlinear methods to better estimate shape parameterization and body composition relative to prior work with linear baselines.

In this work, we trained a deep 3D graph convolutional autoencoder on a diverse sample of full-body 3DO scans. A subset of these scans was captured with paired DXA scans for ground truth body composition training targets. We trained a regression model from extracted 3D graph convolutional features in the deep autoencoder network to DXA body composition variables using a nonlinear Gaussian process regression (GPR). We observed the effect of manipulating depths and dimensions of convolutional features on prediction accuracy and compared nonlinear estimates of 3D reconstruction error and body composition prediction precision and accuracy to results produced by linear methods from prior works. To our knowledge, this is the first application of a 3D graph convolutional autoencoder to body composition prediction. Our results indicate that nonlinear regression using a GPR with a squared dot product kernel provides greater accuracy and precision in body composition estimation on test data than previous linear methods.

## METHODS

This work performs an ablation study between linear and nonlinear methods for shape modeling and body composition estimation of 3D human body shape. We assembled a composite dataset of 3DO human body shape from four independent sources and standardized them to a common topology. We trained a deep 3D autoencoder (3DAE) based on the work of Zhou et al. [18] on the composite dataset at multiple parameter sizes and evaluated its 3D reconstruction error on test data. 10 variables of body composition were studied consistent with prior work with reference values obtained with same-day DXA scans. The variables studied were:

1. Total body fat mass
2. Total body lean mass
3. Visceral fat mass
4. Arm lean mass
5. Leg lean mass
6. Percent body fat
7. Trunk fat mass
8. Trunk lean mass
9. Arm fat Mass
10. Arm lean mass

We trained nonlinear GPR models from 3DAE deep features at multiple scales to predict reference DXA values. We then trained a PCA model as a linear baseline using the same training data and parameter counts as the nonlinear model as an ablation study against prior work. From these linear PCA features we trained linear least squares regression and nonlinear GPR models to create linear-linear and hybrid linear-nonlinear models for DXA body composition prediction. This iterative modeling allowed us to quantify the impact of novel applications of nonlinear methods against linear baselines.

### Experimental Cohort

Four data sets were used for this study: CAESAR [19], Shape Up! Adults (SUA) (NIH R01 DK109008) [14], Shape Up! Kids (SUK) (NIH R01 DK111698) [20], and DFAUST [21]. Table 1 shows how this data was subdivided to train and test our models.

The CAESAR 3DO scan dataset represented 2400 American and Canadian adults aged 18–65 3D scanned in a neutral A-pose, with legs and arms held fully extended and abducted ~ 30 degrees from the midline of the body. Participants were mostly unclothed except for form-fitting gray underwear and a swim cap to standardize hair appearance. Roughly half of the recruited cohort were female. Sex, height, and weight were the only demographic variables used to construct the shape model; no other demographic collected was used in this study. Subjects were scanned on a custom-built Cyberwar WB4 3D scanner.

The SUA and SUK datasets (ClinicalTrials.gov ID NCT03706612 (SUK) and ID NCT03637855 (SUA)) were cross-sectional and stratified by age (SUK: 5–8, 9–12, 13–17 year., SUA: 18–39, 40–59, ≥ 60 year.), ethnicity (non-Hispanic white, non-Hispanic black, Hispanic, Asian, and Native Hawaiian or other Pacific Islander (NHOPI)), sex, and BMI Z-score. Along with extensive demographics, quantitative measures included whole body DXA scans and 3DO scans. We acquired duplicate whole-body DXA scans of each participant on either a Hologic Horizon/A system (UCSF) or a Discovery/A system (PBRC and UHCC) (Hologic Inc., Marlborough, MA, USA). Participants were positioned and scanned according to guidelines specified by the respective system manufacturers. All scans were analyzed at UHCC by a single certified technologist using Hologic Apex version 5.6 with the National Health and Nutrition Examination Survey (NHANES) Body Composition Analysis calibration option disabled. DXA systems quality control was performed by monitoring the weekly values of the Hologic Whole Body Phantom. Two independent 3DO scans were acquired for each participant in up to three scanning devices: Fit3D Proscanner 4.x, Fit3D Inc, Redwood City, CA, USA, Styku S100 4.1, Styku LLC, Los Angeles, CA, USA, and Size Stream SS20, Size Stream, Cary, NC, USA. All 3DO scans were captured in a neutral A-pose that closely mirrored the pose of the CAESAR dataset.

The DFAUST dataset was a 4D capture of human pose. A templated mesh was registered to over 41,000 snapshots from continuous sequence of 3D point clouds (3dMD LLC, Atlanta, GA) of 10 unique individuals performing dynamic movements captured at 60 frames per second. 4264 meshes were reserved for testing and were not used in this study. No clinical data such as body composition was reported.

The DFAUST dataset was used to pretrain the 3DAE model. The pretraining step initialized the network weights to a state similar to the presentation in the original work of Zhou et al. [18] An ensemble dataset of CAESAR, SUA, and SUK was used as training data to finetune the model. During deep network training, a held-out data split, the evaluation set, was reserved to benchmark the performance of the model at the conclusion of each training epoch as determined by its geometric reconstruction loss. 20% of the CAESAR data was reserved for the finetuning evaluation steps (FT eval). To create a standardized benchmark between the methods, the same training and test split in Tian et al. [15] was preserved to investigate the performance of the new deep autoencoder and nonlinear GPR on the same test set. The test set was a random sample of 20% of the available Shape Up! Adults scans.

**Figure 1:**
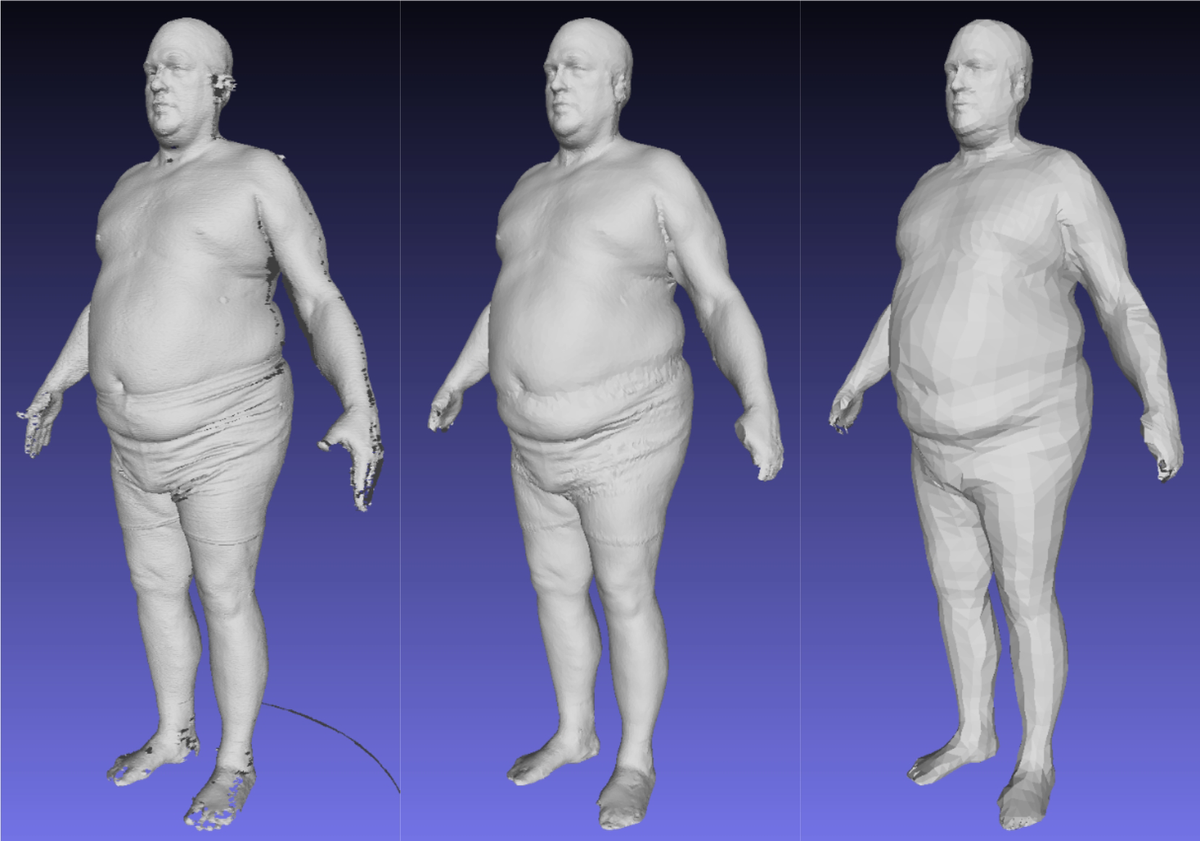
Left: a raw CAESAR scan with unordered vertices and incomplete surfaces. Center: automatic 60k template registration using the methods of Tian et al. Right: SMPL (6890) topology transformation of the 60k fit, created by multiplying the 60k mesh with a sparse nearest-neighbor matrix C and deforming the result to align with the 60k mesh.

To enforce topological consistency between all 3DO meshes, all scans in Table 1 were standardized to a constant topology containing 6890 vertices (referred to as the SMPL [22] topology) except for the DFAUST data, which is already in this format natively. We used the automated template fitting method of Tian et al. [15] and the nonrigid deformation of Allen et al [23] for this process. Implementation details are provided in the Supplementary Materials. A visual example of this process is shown in Fig. 1.

The ensemble 3DO data selected for this study collectively covered many of the limitations inherent to each individual dataset. Clinically sampled, multi-identity datasets like SUA and SUK are custom-made for studies modeling human shape variation and body composition estimation across a diverse, cross-sectional sample of the population. SUA and SUK contain 3D meshes and body composition reference values for adults and kids respectively but are low in participation due to the additional overhead and difficulty of collecting clinical data. CAESAR augments this dataset by doubling the number of single-pose 3DO scans available while almost quadrupling the number of unique individuals represented. However, since there is no clinical data associated with CAESAR, it can only be used to train the 3D shape model and not the mapping to body composition. Only CAESAR scans were held out for model evaluation during the finetuning step of 3DAE training to preserve as many SUA and SUK scans with paired DXA measurements as possible for body composition regression training and testing.

The DFAUST data was different from the other data subsets as it contained very few unique individuals (10, 5 male and 5 female) but the largest number of unique 3DO scans (~ 37,000). This dataset varied pose instead of individual identity to create shape diversity. Modeling shape variation due to posing is not an objective of this study and DFAUST was not used to jointly train the 3DAE shape model with the other datasets. However, DFAUST was very useful in initializing the 3DAE weights to a better-than-random starting state for training on the CAESAR-SUA-SUK ensemble dataset.

### 3D Deep Autoencoder with Graph Convolutional Network

Raw 3DO scans contain potentially hundreds of thousands of unorganized vertices and are not suitable inputs for regression algorithms without first processing then into standardized feature vectors for all dataset members. In this study, we use a 3D graph convolutional neural network adapted from Zhou et al. [18] to perform nonlinear dimensionality reduction and feature extraction on the 3D body shape space. This deep network is a 3D autoencoder that possesses many attributes inherent to deep convolutional neural networks (CNNs). A local kernel operator paired with layered feature pooling and unpooling, equivalent to multilevel filters in 2D image CNNs, gives this method local feature sensitivity while enabling the representation of nonlinear relationships between the latent encoding and the decoded shape. For a 3D autoencoder, the inputs are the 3D mesh vertex coordinates represented as 6890×3 sized tensors. The loss is a geometric mean absolute error (MAE) loss minimizing the reconstruction error against the original input coordinates.

**Figure 2:**
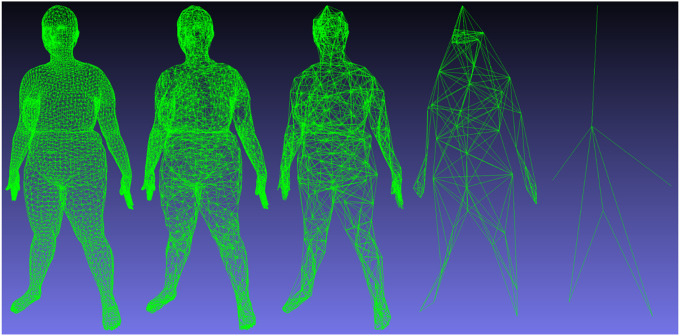
Successive convolutional downsampling of a human mesh with a radius and stride of 2. The images represent in order the input mesh (6890×3) and the intermediate graph topologies containing (# vertices × # channels): (6890×16), (1925×32), (400×64), (54×128), (7 × f). The final layer is the bottleneck layer, whose channel count f is variable in our experiments.

Unlike image convolution on a regular square grid, the 3D mesh graph is irregular with varying connectivity. This requires some architectural modifications in the network to apply convolutional operations to human body scans. Our chosen implementation of a 3DAE assumes topological consistency of all mesh inputs. This simplifies the network design by allowing us to determine the spatial down sampling and upsampling operations once per mesh template in a preprocessing step. We defined both the convolutional kernel radius and the spatial kernel stride as two to be consistent with Zhou et al. A visualization of the precomputed graph down sampling for each layer of the autoencoder is shown in Fig. 2. Additional implementation details are written in the Supplementary Materials and in Zhou et al.

Data paucity was one of the primary reasons for using linear algorithms rather than a deep network in prior work as 3DO data paired with ground truth DXA measurements are absent in the literature outside of the limited SUA and SUK collections. We initialized our model with the DFAUST dataset as augmentation data to mitigate the lack of data availability. We pretrained our 3DAE on DFAUST for 200 epochs, then trained on a composite of SUA, SUK, and CAESAR for an additional 400 epochs excluding DFAUST. We trained 3DAE models bottleneck channel depths of 7, 43, 90, and 601. With 7 nodes at the bottleneck layer, these channel depths defined total latent feature vector sizes of 49, 301, and 630, and 4284 as shown in Fig. 1. All models were trained with a kernel basis size of *M* = 37. The learning rate was set to 1e-4. Training batch size was set to at 16. (See Zhou et al. for implementation details) All model variations had the same architecture and training data aside from the bottleneck layer.

### Learning a nonlinear transfer function with GPR

For body composition analysis, we performed nonlinear Gaussian process regression between the features extracted from the SUA dataset and their paired DXA measurements. Prior work on estimating DXA body composition from 3D body shape used least squares linear regression, which imposed a restrictive assumption of linearity on the relationship between body shape variables and body composition. GPR was previously used in Wang et al. [25] for visceral adipose tissue estimation from body circumferences and is a nonlinear, probabilistic generalization of linear regression that relaxes the imposition of linearity on the function between body shape features and body composition. GPR is more generalized than linear regression but not as unrestricted as a multi-layer perceptron network (MLP). The limited number of data observations in our dataset made GPR a more appropriate regression method than MLPs while still relaxing tight assumptions on the function shape.

We trained a GPR model to learn a nonlinear mapping between the encoded latent vectors of the training data and the DXA measurements for 10 body composition measures. We chose a squared dot product kernel for GPR under the assumption that relationships between body shape features and body composition were nonlinear but monotonic in both the first and second derivatives. Our assumption of monotonicity between body shape and body composition is consistent with the observation that variables such as visceral fat and percent fat are positively correlated with anthropometric measurements such as waist circumference and body volume [16]. We experimented with other kernels such as the radial basis function (RBF) and higher powers of dot product kernels and found they performed worse than a squared dot product kernel. We present results for the squared dot product kernel in this paper. We trained GPR models for male and female participants separately.

GPR derivation details can be referenced in the Appendix and in greater detail in [26] and [27]. We implemented GPR using scikit-learn [28]. We concatenated [height, weight, age] of each participant to the feature vector input to GPR in accordance with the procedures established in previous work. To comprehensively search the feature representation space across multiple scales for the best predictive inputs, we performed GPR on all intermediate feature layers of the 3DAE shape model to predict body composition targets. For each of the four levels of deep features shown in Fig. 2, we performed GPR to body composition targets. We reported the prediction accuracies for the bottleneck layer containing 4284 (7×612) features and for the feature layer producing the lowest RMSE in GPR prediction.

### Comprehensive ablation trials vs. linear methods via model permutations

PCA is a common linear approach to dimensionality reduction for 3D human body shape due to the widespread adoption of methods such as SMPL [22] and was the shape modeling method used in prior work on 3DO body composition estimation. PCA is a deterministic linear operation with a globally optimal solution and produces feature vectors over a space of orthogonal components, making it well-behaved even for datasets containing just tens to hundreds of scans [15, 23].

To test the comparative performance of new nonlinear models against linear baselines established in previous work, we trained a PCA model using the same 4286 training meshes of the 3DAE. DFAUST was not used for training PCA shape models. Although the test set membership of this study is the same as that of Tian et al (2022) [15], the training set in this work is greatly expanded. Recreating PCA models with consistent data membership allowed us to isolate the effects of deep 3DAE shape encoding and nonlinear GPR prediction relative to a baseline of PCA and ordinary least squares (OLS) on the same data. Due to the predefined downsampling of the 6890-vertex mesh topology shown in Fig. 2, the bottleneck layer of the 3DAE must be a multiple of 7. We set the maximum bottleneck (latent code) size of our 3DAE to 4284 as it was the closest multiple of 7 to 4286, the maximum number of possible PCA components (corresponding to the number of meshes in the training set). While the 3DAE bottleneck layer dimension could be increased to an arbitrarily high number, resulting in lower reconstruction error, there would not be an equivalent linear PCA model to function as a comparative baseline.

To further test our hypothesis regarding the better prediction accuracy of nonlinear GPR relative to OLS, we trained a GPR model using the same PCA basis from Tian et al. (2022) [15], labeled PCA-GPR M391/F457 which indicates 391 and 457 components for males and females respectively.

### Statistical Analysis

We measured the geometric reconstruction accuracy of both linear and nonlinear shape models at different model sizes to assess the marginal contribution of nonlinear autoencoders to shape modeling accuracy relative to a linear PCA method used in prior work. We then iterated through different model permutations consisting of feature extraction with both linear and nonlinear shape models followed by both linear and nonlinear regression to DXA body composition measurements. Model permutations demonstrated the marginal contributions of GPR and 3DAE to the precision and accuracy of body composition prediction relative to the baselines established by linear algorithms used in prior work.

We compared 3D geometric reconstruction error between the PCA baseline and 3DAE shape models at four model sizes as the per-vertex mean absolute error (MAE). We compared the accuracy of body composition estimation for four model permutations: the GPR baseline estimated using only demographic features [height, weight, age] with no shape information, the linear baseline consisting of ordinary least squares regression from PCA shape features (PCA-OLS), hybrid consisting of GPR using PCA shape features (PCA-GPR), and fully nonlinear GPR from deep shape features (3DAE-GPR). We quantified estimation error with root-mean-squared-error (RMSE) relative to reference DXA measurements and plotted the normalized RMSE of each predicted metric as a percentage of the PCA-OLS fully linear baseline. The coefficient of determination (R^2^) for agreement to DXA was assessed for the 3DAE-GPR model.

We predicted body composition on test-retest data pairs to compare the precision of our model permutations to prior work and to DXA scanners using the coefficient of variation (%CV) of visceral fat and the repeat RMSE of percent fat. We compared the precision of 3DAE-GPR at two model sizes against the PCA-GPR and PCA-OLS permutations, trained and tested on the same training data and test-retest pairs. For 3DAE-GPR, we trained the GPR component on the bottleneck layer (301 and 4284 features for the small and large model sizes respectively) to enforce consistency with the parameter count of the PCA permutations.

We compared the accuracy of our best model performance to a comprehensive list of prior work using percent fat and visceral fat prediction as the benchmark and showed that we can achieve state of the art results on 3DO body composition prediction using deep convolutional feature extraction and nonlinear regression.

We conducted ablation studies to evaluate the effects of skipping model training steps or withholding training data on geometric reconstruction accuracy and body composition prediction accuracy to confirm that all training procedures and all subsets of the data described in the method positively contributed to the accuracy of our results. We recorded the reconstruction accuracy of the 3DAE using a random initialization without the DFAUST pretrained initialization. We then tested the inverse data withholding condition by training a 3DAE on DFAUST only with no further finetuning with our multi-identity ensemble dataset on SUA test data. We withheld CAESAR, SUK and both CAESAR and SUK data from the training sets to test if dissimilarities between data subsets may have reduced reconstruction or regression performance. We tested the geometric reconstruction accuracy and the body composition prediction accuracy for each exclusion scenario. Ablation trials were tested on a *d* = 301 sized bottleneck network due to its much lower training time.

## RESULTS

The Shape Up! Adults study population has been previously described [15] and summarized in Table S1 in the Supplementary Material. Only this subset of the total data was used for body composition regression training and testing.

Geometric reconstruction error for both 3DAE and PCA shape models of four increasing sizes are shown in Table 3. The dimensionality *d* represents either the number of PCA coefficients used to parameterize shape (linear model) or the number of latent variables in the bottleneck layer of the 3DAE (nonlinear model) connecting the encoder module to the symmetric decoder. Reconstruction error was calculated as the geometric mean absolute error (MAE) between original and reconstructed vertex 3D positions. As expected, larger models were able to reconstruct the test data with lower error. Both linear and deep methods are comparable in terms of geometric reconstruction accuracy for the first three model sizes. PCA achieved lower geometric reconstruction error at the highest parameter count while 3DAE reconstruction error leveled off at just above 2mm MAE.

**Figure 3:**
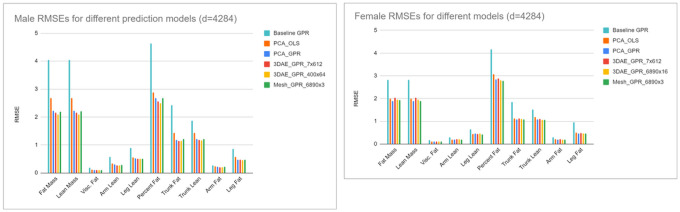
RMSE prediction errors for different model permutations. Every column represents exactly one modification to the pipeline parameters from the previous column (i.e. OLS to GPR) excluding the baseline column. RMSE is shown in kg except for PFAT, which is expressed as a percentage. Baseline is GPR prediction from known priors [height, weight, age] only. PCA_OLS is linear regression from linear PCA features. This result was identical to OLS from the 3DO vertex coordinates. PCA_GPR is nonlinear GPR prediction from linear PCA features. Mesh_GPR is GPR prediction from 3DO vertex coordinates with no feature extraction. 3DAE_GPR_X is the most accurate GPR model trained from any feature layer of the 3DAE network with the feature dimension indicated as X.

Figure 3 depicts the body composition prediction results from *d*=4284 sized models using different permutations of linear and nonlinear shape feature extraction and regression methods. Excluding the baseline column, each subsequent column represents a change of exactly one model parameter holding all others constant; i.e. OLS to GPR, PCA to 3DAE, 4824 total parameters to 400×64 parameters. All model permutations were trained and tested on the exact same mesh data.

The baseline GPR model accuracy is the RMSE resulting from a regression using only known features [height, weight, age] without any conditioning on the 3DO shape features as was previously done in [15] and [29]. This RMSE was higher than any subsequent regression model using shape features as input. The reduction of prediction error when shape information is introduced demonstrated that 3DO shape is a useful signal for body composition prediction even when nonlinear regression algorithms are employed.

PCA-OLS is equivalent to the linear methods of prior work. This is a linear regression model for body composition prediction taking PCA features as inputs. The PCA model had 4284 parameters to stay exactly consistent with the dimensionality the 3DAE.

PCA-GPR represents a hybrid pipeline predicting body composition with nonlinear GPR from linear PCA features. Nonlinear regression from linear shape features achieved lower RMSE on every predicted metric relative to linear regression except arm lean on females (which was equal) and leg lean on females (which was higher by 0.02 kg, or 5%). These results indicated that GPR was a more accurate regression method than OLS when all other factors were held constant for most body composition targets.

3DAE-GPR represents the fully nonlinear pipeline where body composition is predicted from GPR using 3DAE deep features as inputs. The results for both the bottleneck layer (7×612) and the layer with the lowest RMSE are shown. The third layer (dimensions 400×64) was the most accurate feature layer for males and the first layer (dimensions 6890×16) was the best for females. In males, 3DAE feature extraction lowered RMSE relative to the previous model permutations, but in females no level of feature extraction outperformed GPR on the original mesh coordinates. This result suggests the features extracted from female meshes were less informative relative to male features or were highly correlated regardless of the method and model size. GPR always improves accuracy relative to OLS, but 3DAE feature extraction only improved accuracy for males.

OLS regression to body composition with 3DAE features resulted in very low correlations with DXA due to the nonlinearity of the deep features and was not reported. We also tested concatenating all feature layers into a single multi-scale feature vector for GPR regression but did not achieve lower errors in doing so.

**Figure 4:**
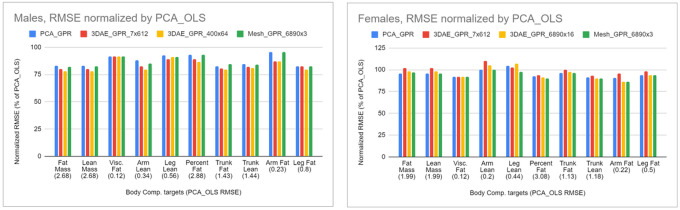
Normalized RMSE values from Figure 3 as a percentage of the linear baseline (PCA_OLS), shown in parentheses below every body composition metric.

We rescaled the charts in Figure 3 by normalizing each column by the RMSE of the fully linear PCA_OLS model and plotted the values in Figure 4 for visual clarity. In males, all subsequent model permutations had RMSEs less than that of PCA_OLS (indicated by a normalized RMSE of 100%). For females, only leg lean increased in RMSE when moving from PCA_OLS to PCA_GPR. However, as previously observed most RMSEs increased in females when incrementing PCA to 3DAE. When comparing nonlinear methods to linear methods, GPR is more accurate for body composition prediction in most metrics, but 3DAE is not always a better feature extraction method for body composition prediction from shape relative to linear PCA in females.

Test-retest precision of percent fat and visceral fat estimation is shown in Table 3. For visceral fat mass precision, Coefficient of Variation (%CV) was calculated according to the definition in Glüer et al. [30] between two scans of the same individual in the test set taken on the same day. RMSE between trial 1 and trial 2 was shown for percent fat precision as the %CV of a percentage measurement is not used in convention.

GPR was more precise on retests than OLS as shown by the PCA-OLS versus PCA-GPR trials, where the latter resulted in up to a 30% decrease in precision error. 3DAE also decreased precision error relative to PCA in both sexes, as illustrated by the 3DAE-GPR versus PCA-GPR trials. 3DAE coupled with nonlinear GPR had the highest precision. Compared to DXA, percent fat precision error for 3DAE-GPR was roughly twice as high. However, visceral fat precision was lower than DXA, indicating the 3DAE model paired with GPR prediction is much more reliable on retest accuracy than prior work. The larger *d*=4284 3DAE model was comparable in precision to the smaller model. 3DAE was more precise than PCA, and GPR was more precise than OLS.

Comparisons of our 3DAE-GPR models against prior work are shown in Table 4 using the d=4284 latent size model and Gaussian process regression with the best performing feature layer (third for males, first for females). Percent fat (PFAT) and visceral fat mass (VFAT) were selected as the comparative target variable as they achieved the lowest accuracy in previous works based on linear models.

GPR using the exact same PCA features of Tian et al. (PCA-GPR M391/F457) lowered the RMSE from OLS for percent fat in both sexes and in female visceral fat but increased it slightly in male visceral fat by 8%. The models presented in [15] were built on a dataset containing an order of magnitude fewer members than what we presented in this work. These results suggest linear pipelines may be more competitive with nonlinear methods when training data quantity is more limited. We included RMSEs for our best performing, maximum size PCA model (PCA-GPR 4284) to demonstrate that increased parameter count does not cause overfitting and degrade accuracy relative to a sparser model.

Our fully nonlinear model, 3DAE-GPR, produced the lowest error on visceral fat and percent fat estimation compared to all prior work on 3DO body composition estimation (bolded). We note that compared to Tian et al (2022), our best 3DAE-GPR model achieved lower RMSE on all of the 10 body composition metrics measured in Figure 4, shown in Supplementary Table 2. However, predicted metrics other than percent fat and visceral fat already showed high correlation with DXA in prior work using linear methods. Thus, we focused the comparison to prior work in Table 4 on the metabolically significant and previously underperforming predictions of percent fat and visceral fat. The test set used in this work was held the same as [15]. However, the training dataset was greatly expanded in size and scope relative to prior works.

### Ablation results

**Figure 5:**
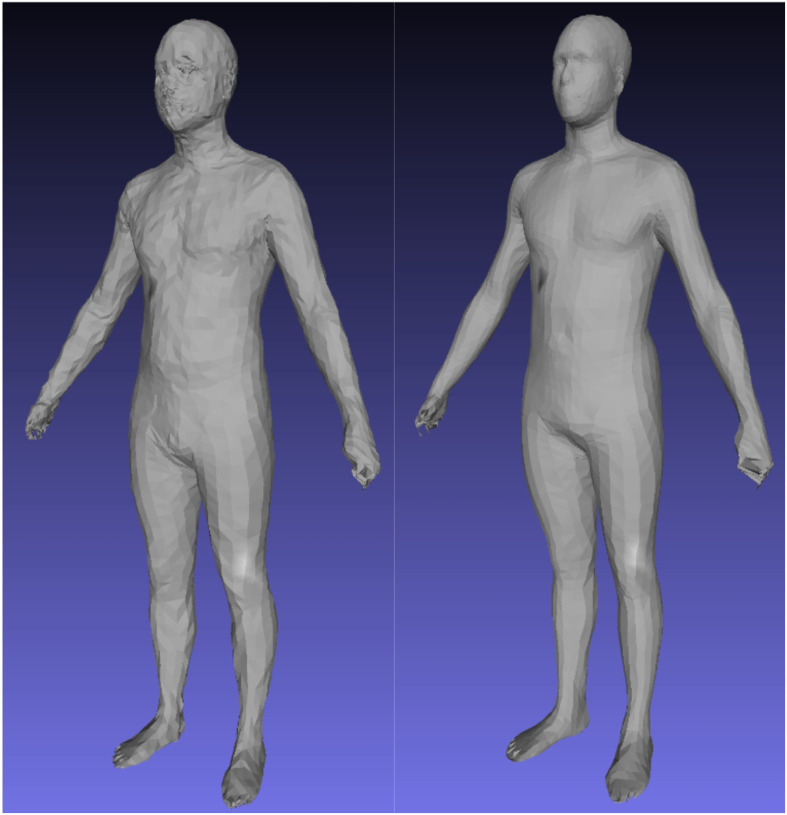
Pretraining on 40,000 DFAUST meshes improves reconstruction fidelity in single pose, multi-identity data. Left: Evaluation set mesh reconstruction after training 400 epochs from random initialization. Test set reconstruction error was 22.7mm. Right: Same mesh reconstruction using a model pretrained first on 200 epochs with DFAUST only followed by 400 epochs of finetuning. Test set reconstruction error was 2.0mm.

Figure 5 shows a mesh reconstruction using a *d*=4284 model trained with 400 epochs on the finetuning ensemble training data from 1) a random initialization state and 2) from a pretrained initialization trained with 200 epochs using DFAUST data only. The model trained from a random initialization achieved 22.7mm MAE on test data reconstruction, more than 10x the error of the error show in Table 3. The pretraining steps using 40,000 DFAUST meshes was essential for creating an accurate shape model using a nonlinear 3DAE.

**Figure 6:**
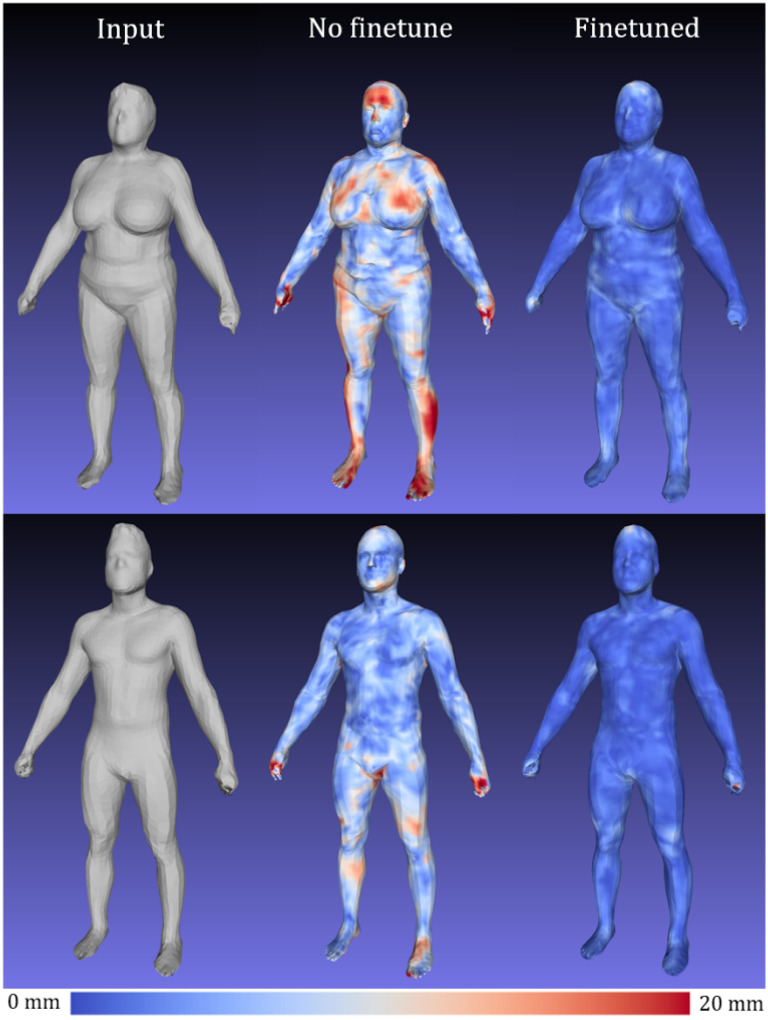
Finetune training on 4286-member multi-identity ensemble dataset reduces reconstruction error on test set meshes relative to 3DAE models trained on DFAUST only. Left: input 3DO test mesh. Center: reconstruction error heatmap using model state following 200 epochs of pretraining on DFAUST only. Test set MAE was 8.7mm. Right: heatmap on same test mesh after an additional 400 epochs of finetuning on multi-identity, single pose ensemble dataset. Error heatmap is in the range of [0,20] mm.

Figure 6 shows visualizations of geometric reconstruction error as heat maps for a male and female subject before and after fine-tuning the *d*=4284 3DAE model with high resolution Shape Up! and CAESAR data. Without fine-tuning, the 3DAE model was equivalent to the work presented in Zhou et al. [18] trained exclusively on DFAUST data and achieved a 3D reconstruction error of 8.7 mm, as opposed to the 2.0 mm shown in Table 2 for the finetuned model. This model generalized very poorly to unseen scans of many unique individuals such as in Shape Up!, as DFAUST only contained 10 unique individuals captured in thousands of different poses. A 3DAE model for clinical machine learning applications needs to generalize well to any individual scanned from the general population in a neutral pose. Our fine-tuned model represented the non-rigid, identity-dependent deformations of unique individuals much more accurately.

Withholding non-SUA data from 3DAE training did not improve 3D reconstruction error on SUA test meshes. In all three ablation trials, reconstruction accuracy on the same SUA test set (2.58mm, 2.71 mm, 2.76mm for removing only SUK, only CAESAR, and both, respectively) was worse than the model trained on all data combined (reconstruction accuracy of 2.57) in Table 3. This ablation result validates the inclusion of a diverse dataset across multiple collection protocols.

GPR models trained on the 3DAE model excluding SUK data showed 1 % difference in RMSE in both directions. Not all target variables were uniformly lower in error when including versus excluding SUK. The variation could be attributed to noise and does not justify excluding SUK. Excluding CAESAR data from the training scans had similarly negligible effects on male prediction accuracy but increased female RMSEs without exception by up to 4%. Excluding both SUK and CAESAR produced results similar to the case where only CAESAR was excluded, but with even higher errors in females. Overall, we found that including all available 3D mesh data when training shape and regression models produced the lowest errors for 3D reconstruction and body composition prediction.

## DISCUSSION

This study showed three primary findings. First, GPR generally improves body composition prediction accuracy and precision relative to linear regression regardless of whether the feature domain is linear (PCA) or nonlinear (3DAE). By comprehensively exploring model permutations and changing exactly one variable at each iteration, we were able to demonstrate that nonlinear regression with GPR performed better than OLS for every body composition target except female leg lean. Second, 3DAE feature extraction improved body composition prediction for males. However, deep features were not more informative than the unencoded mesh geometry in females and did not outperform PCA in geometric reconstruction at high parameter counts. Third, both 3DAE and GPR achieved higher precision than their linear counterparts in both males and females when measured on percent fat and visceral fat mass.

A combination of an expanded and diversified training set with deep feature extraction and nonlinear GPR prediction allowed us to build an end-to-end model that outperformed all prior works on body composition prediction accuracy. The improved accuracy and precision demonstrated here builds on our prior work [14, 15] and further establishes 3DO as a reliable and accessible clinical tool for the assessment of body composition. Such a tool has promising clinical implications for the management of chronic disease, where body composition has known associations with morbidity and mortality.

To our knowledge this was the first application of deep nonlinear autoencoder networks to 3DO body composition estimation. We explicitly chose a spatial graph convolutional network for our autoencoder approach. Other autoencoder approaches, such as implicit surface encoding [32], non-convolutional variational architectures [33], and spectral graph convolution [34] have been used to study body shape, but without associations to clinical outcomes. Future work may find that these other approaches have advantages to the work presented here.

We hypothesized that lower geometric reconstruction error in a shape autoencoder model was correlated with higher body composition prediction accuracy during regression from the extracted features. However, although a linear PCA model of size *d* = 4284 produced the lowest shape reconstruction error, it did not outperform 3DAE on body composition prediction accuracy or precision. Geometric reconstruction accuracy may be affected by shape deformations irrelevant to body composition variation such as pose or face detail. PCA may be outperforming 3DAE in shape reconstruction at high parameter counts due to 3DAE potentially learning redundant and correlated features when the model size is large. Unlike 3DAE, PCA is guaranteed to learn uncorrelated, orthogonal features due to its mathematical construction. Future work should investigate the relationship between geometric reconstruction accuracy and body composition prediction accuracy by controlling for uncorrelated geometric variations and using different autoencoder architectures.

PCA’s high performance on shape reconstruction validate the methods of past work built on PCA models with linear regression [14, 15, 20] despite the restrictive linear assumptions of the algorithm. Prior work restricted the shape model and body composition prediction features to a sparse subset of the total number of PCA components to avoid overfitting during regression. Our extensive testing with different model sizes and permutations does not support the assumption that large parameter counts overfit on test data. PCA-GPR model using 4284 components achieved lower RMSEs on percent fat and visceral fat on both males and females relative to models trained with 391 or 457 features. Restricting shape and regression models to the first *n* components that describe 95% or 99% of the shape variance may not be justified in future work as it potentially handicaps prediction accuracies unnecessarily.

Our chosen autoencoder architecture does not explicitly disentangle pose deformation from identity-dependent deformations, such as in Wong et al. [31] or Jiang et al [33]. Factoring out slight pose variation across our dataset may improve the reconstruction accuracy and regression accuracy of our method. Increased sensitivity to small changes in body shape may allow our method to improve monitoring body composition change over time in the same individual [35]. As our dataset was transformed to adopt the mesh topology of SMPL [22] while preserving the geometric surface detail of the original 3DO scans, it may be straightforward in future work to “unpose” every mesh in our 4286-member ensemble dataset to a neutral T-pose like that of Wong et al. using the skinning weights and joint position definitions of the SMPL template. The accuracy of this procedure will depend on how anatomically consistent the template correspondences are between our dataset and the meshes in Loper et al.

Future work can explore the impact of different nonlinear regression methods on body composition prediction accuracy. GPR regularized the shape of the regression function to its kernel. A less restricted but more flexible regression method such as a multi-layer perceptron (MLP) may achieve better prediction error with proper regularization; however, deeper models run the risk of overfitting to the training data especially if the latent parameter count is large. We observed that GPR using a radial basis function kernel (RBF) already exhibits symptoms of overfitting, showing zero error on the training data but greatly increased error on the test data.

Like prior works using linear models, this work separately trains a shape feature extractor followed by a body composition regression from features. Future work may integrate the current two-step pipeline into a single end-to-end deep network. This can be achieved by connecting the 3DAE encoder layers directly to a neural network regression model that targets body composition as its output instead of self-reconstruction. This end-to-end model can be initialized to state reported in this work by importing our trained 3DAE weights into the encoder of the network and the weights of a separately trained regression model into the neural network regression layers. GPR may be recreated as a neural network in PyTorch with GPyTorch [36]. This implementation could allow the combined network to achieve greater accuracy by starting from the state presented in this work and further optimizing all features to target body composition prediction with no intermediate objectives.

**Figure 7:**
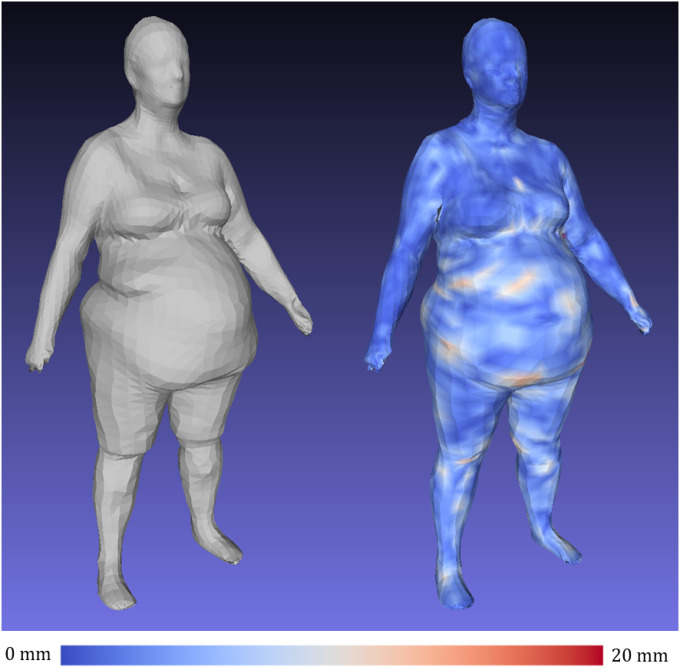
Left: an input test mesh with high BMI; right, the heat map of the reconstruction showing where the greatest errors occurred. The numerous folds in the abdomen of this individual (47.3% body fat) were unique to only a few scans in the dataset and were possibly insufficiently modeled in the latent encoding.

Our 3D deep shape model was trained on the largest collection of high quality, multi-identity 3DO scan data currently available, spanning CAESAR, SUA, SUK, and DFAUST over more than a 20-year period. However, with 2,900 unique individuals, this dataset is still orders of magnitude smaller than those used in analogous networks for deep 2D image learning. This restriction on dataset size and population variation sampling density can create gaps in our model that exhibit low reconstruction and prediction accuracy where training data was not available or under sampled, especially at the extremes of body shapes as shown in Fig. 7. Ongoing data collection of high resolution 3DO scans will improve the reconstruction accuracy of our method. Improvement of the nonlinear regression model will require additional paired DXA scans.

**Figure 8:**
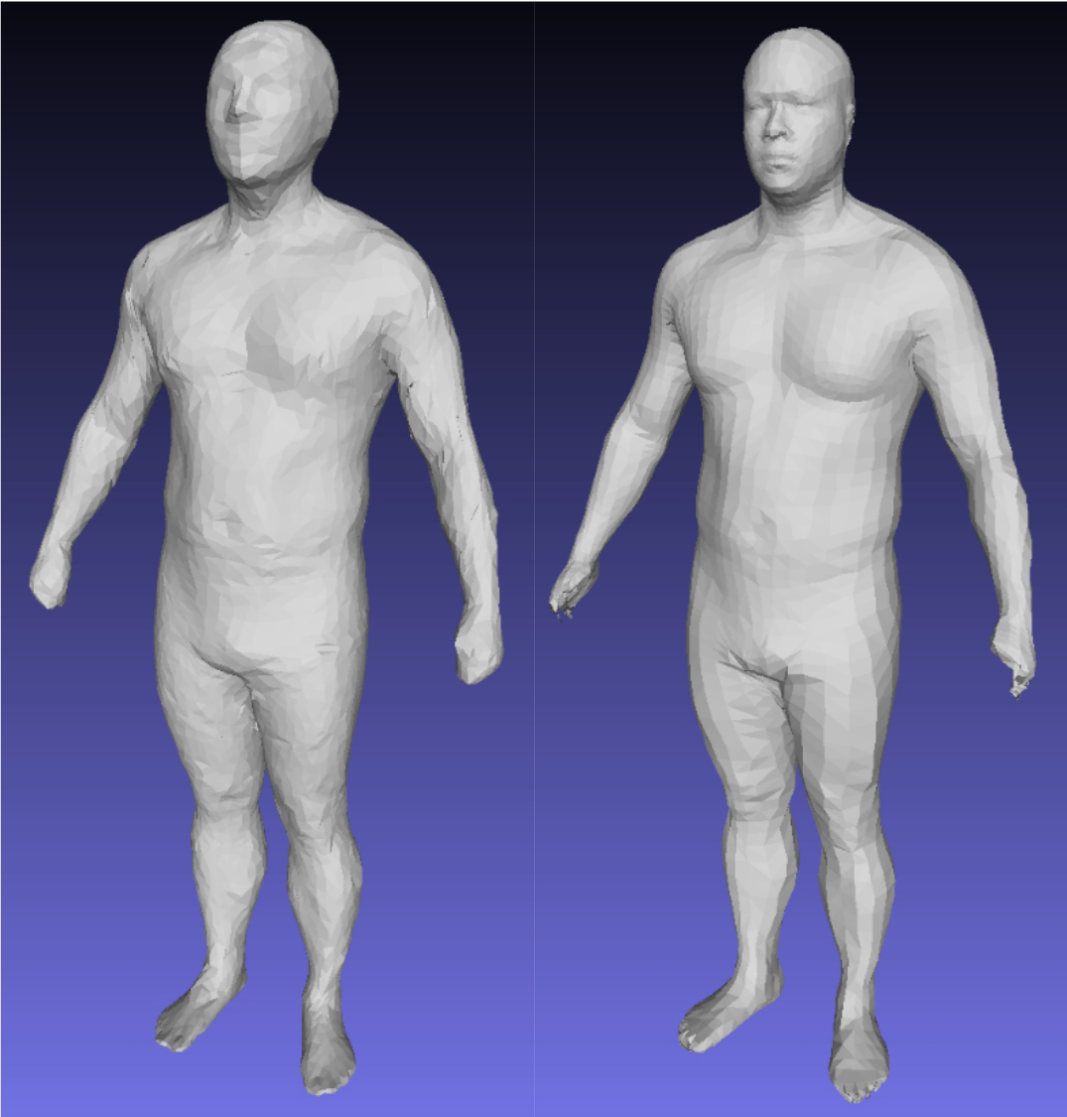
MPI CAESAR mesh (Left) compared to our remeshing of the same individual (Right). The MPI shape was projected onto a linear PCA basis and lost considerable identifying detail.

The CAESAR data used in this work was built from the original 3D scan data collected by Robinette et al. [19] and not on a derived reconstruction such as the MPI CAESAR dataset produced by Pishchulin et al [37]. The MPI dataset is a template standardization of CAESAR using a limited number of PCA features for reconstruction. The resulting shapes are linearly projected compressions of the original 3D scan data and do not preserve original high-resolution detail well, as shown in Fig. 8. Including these shapes into our training database would bias our deep nonlinear model towards an approximation of a linear solution. Our remeshing of the CAESAR scans into the SMPL format includes a nonrigid surface-to-surface deformation that produced templated meshes consistent with the original scan geometry and did not constrain the training data to projections onto a linear subspace. Future work studying human shape variation as a function of varying identity rather than pose may build upon our results by utilizing the higher fidelity standardized templates of our dataset rather than training on potentially degraded 3D shape geometry.

## CONCLUSION

A comprehensive comparison of nonlinear methods for shape feature extraction and body composition regression against previous linear algorithms showed that nonlinear GPR improved body composition prediction accuracy and precision relative to linear regression for 10 metrics of body composition in males and for 9 in females. Nonlinear GPR produced up to 20% reduction in prediction error and up to 30% increase in precision over linear regression for both sexes in 10 tested body composition variables. Relative to linear PCA feature computation, feature extraction with a deep 3D autoencoder provided marginal improvements to prediction accuracy for males but did not supersede the performance of GPR on raw mesh vertex positions for females. Deep shape features produced 6–8% reduction in prediction error over linear PCA features for males. However, deep 3DAE features reduced precision error between 4–14% relative to linear features with all other variables held equal. Our best performing nonlinear pipeline using 3DAE-GPR outperformed prior works on body composition prediction accuracy for all metrics. Precision error of our method is within 1–2x that of DXA, the gold standard for compartmental body composition measurement. Agreement with DXA as measured by R^2^ values were greater than or equal to 0.86 for all predicted metrics. These findings improve the clinical utility of 3DO as accurate and accessible tool for the assessment of body composition. Future work in this space should explore the effects of disentangling pose variations from 3DO scans along with different deep network architectures such as variational latent encodings, end-to-end deep models, and predicting longitudinal change over time in individual subjects.

## Figures and Tables

**Table 1:** 3D scan count and unique individual representation for the pretrain, fmetune, evaluation, and test sets. The pretraining step used only DFAUST data and included its own evaluation split consisting of 20% of the DFAUST scans. The finetune step was an iterative training step performed using only CAESAR, SUA, and SUK data starting with the network weights determined by the pretraining step. The evaluation set was used to determine the best performing model state to save without causing overfitting to the training data. The test set was kept the same as prior work to create controlled comparisons.

Dataset	Total 3D scans / Total unique individuals			
	Pretraining	Finetuning	FT Eval	Test
DFAUST	36,956 / 10	0	0	0
CAESAR	0	2140 / 2014	462 / 462	0
Shape Up! Adults	0	1500 / 542	0	424 / 336
Shape Up! Kids	0	646 / 200	0	0
**Total**	-	4286 / 2882	-	-

**Table 2.** Per-vertex reconstruction error measured as mean absolute error (MAE) between input and decoded meshes for held-out test meshes from Shape Up! Adults (n=424). The dimensionality (d) represents the number of latent layer variables in the autoencoder bottleneck or the number of principal components used for a linear shape space reconstruction.

	*D*=49	*d*=301	*d*=630	*d*=4284
3DAE MAE (mm)	5.16	2.57	2.18	2.02
PCA MAE (mm)	5.26	2.41	2.23	1.0

**Table 3.** Test-retest precision between repeat 3DO scan pairs of each participant in the SUA test set on the same scan device. 3DAE models were benchmarked with GPR trained from their bottleneck layers to standardize the comparison between model sizes. 3DAE was more precise than PCA, and GPR was more precise than OLS.

*Visceral fat % CV*	3DAE-GPR 301	3DAE-GPR 4284	PCA-GPR 4284	PCA-OLS 4284	Tian et al. 2022 [15]	DXA (Criterion)
Males (n = 143)	4.4%	5.0%	5.2%	7.2%	8.8%	4.8%
Females (n=199)	5.5%	5.5%	6.2%	8.6%	12.3%	6.3%
*Percent fat precision RMSE*	3DAE-GPR 301	3DAE-GPR 4284	PCA-GPR 4284	PCA-OLS 4284	Tian et al. 2022 [15]	DXA (Criterion)
Males (n = 143)	1.0%	0.9%	1.1%	1.6%	1.9%	0.5%
Females (n=199)	1.2%	1.2%	1.4%	2.0%	2.9%	0.5%

**Table 4.** Root-mean-squared errors (RMSE) for predicted percent fat (PFAT) and visceral fat (VFAT) of all current 3D-optical body composition prediction literature on Shape Up! Adults compared to the 3DAE-GPR prediction of the d=301 and d=4284 models using the most accurate feature layer identified in Figure 3. Best performing values are bolded.

Paper	*N* test meshes	PFAT RMSE (%)	VFAT RMSE (kg)
Ng et al (2019) Anthro only	M: 177	M: 4.03	M: 0.15
	F: 230	F: 3.99	F: 0.14
Ng et al (2019) [15]	M: 177	M: 3.55	M: 0.14
	F: 230	F: 3.88	F: 0.13
Tian et al (2020) [28]	M: 31	M: 3.90	M: 0.15
	F: 39	F: 3.29	F: 0.17
Wong et al (2021) [31]	M: 159	M: 2.73	M: 0.13
	F: 202	F: 3.46	F: 0.13
Tian et al (2022) [15]	M: 182	M: 3.24	M: 0.12
	F: 248	F: 4.22	F: 0.14
PCA-GPR M391/F457 [15]	M: 182	M: 2.79	M: 0.13
	F: 248	F: 3.09	F: 0.12
PCA-GPR 4284	M: 181	M: 2.68	**M: 0.11**
	F: 239	F: 2.85	**F: 0.11**
3DAE-GPR 4284	M: 181	**M: 2.50**	**M: 0.11**
	F: 239	**F: 2.81**	**F: 0.11**

## Data Availability

The datasets generated and/or analyzed during the current study are not publicly available due to the inclusion of protected health information (PHI) but are available from the corresponding author on reasonable request. The underlying code for this study [and training/validation datasets] is not publicly available but may be made available to qualified researchers on reasonable request from the corresponding author.

## Abbreviations

3DO: 3Dimensional Optical
60k: 60001 vertex mesh template
BMI: body mass index
DXA: dual X ray absorptiometry
PCA: principal component analysis
